# ﻿Three novel species of *Aquapteridospora* (Distoseptisporales, Aquapteridosporaceae) from freshwater habitats in Tibetan Plateau, China

**DOI:** 10.3897/mycokeys.102.112905

**Published:** 2024-02-23

**Authors:** Rong-Ju Xu, Jun-Fu Li, De-Qun Zhou, Saranyaphat Boonmee, Qi Zhao, Ya-Ya Chen

**Affiliations:** 1 Key Laboratory for Plant Diversity and Biogeography of East Asia, Yunnan Key Laboratory of Fungal Diversity and Green Development, Kunming Institute of Botany, Chinese Academy of Sciences, Kunming, Yunnan 650201, China; 2 Guizhou Provincial Institute of Crop Germplasm Resources, Guiyang 550006, China; 3 School of Science, Mae Fah Luang University, Chiang Rai 57100, Thailand; 4 Center of Excellence in Fungal Research, Mae Fah Luang University, Chiang Rai 57100, Thailand; 5 Department of Economic Plants and Biotechnology, Yunnan Key Laboratory for Wild Plant Resources, Centre for Mountain Futures (CMF), Kunming Institute of Botany, Kunming, Yunnan 650201, China; 6 Institute of Fanjing Mountain National Park, Tongren University, Guizhou 554300, China

**Keywords:** 3 new taxa, freshwater fungi, morphology, phylogeny, Sordariomycetes, taxonomy

## Abstract

During an investigation of lignicolous freshwater fungi in the Tibetan Plateau, three *Aquapteridospora* taxa were collected from freshwater habitats in Xizang, China. The new species possess polyblastic, sympodial, denticles conidiogenous cells and fusiform, septate, with or without sheath conidial, that fit within the generic concept of *Aquapteridospora*, and multi-gene phylogeny placed these species within *Aquapteridospora*. Detailed morphological observations clearly demarcate three of these from extant species and are hence described as new taxa. The multi-gene phylogeny of the combined LSU, *TEF*1-α, and ITS sequence data to infer phylogenetic relationships and discuss phylogenetic affinities with morphologically similar species. Based on morphological characteristics and phylogenetic analyses, three new species *viz. A.linzhiensis*, *A.yadongensis*, and *A.submersa* are introduced. Details of asexual morphs are described, and justifications for establishing these new species are also provided in this study.

## ﻿Introduction

Freshwater ascomycetes are the ecological groups that occur saprobically on submerged or partially submerged plant substrates in aquatic habitats (Shearer 1993). Lignicolous freshwater fungi represent a highly diverse taxonomic group with a substantial population. These fungi play a pivotal role in the transfer of nutrients and the flow of energy between trophic levels in the food chain. They achieve this by breaking down complex organic compounds into simpler inorganic materials derived from dead flora and fauna (Krauss et al. 2011; Sridhar et al. 2013; Wurzbacher et al. 2014; Tsui et al. 2016). Recent research showed that lignicolous freshwater fungi comprise a diverse taxonomic assemblage, with more than 3,870 species listed (Calabon et al. 2022). Among them, most are in the classes Dothideomycetes and Sordariomycetes (Hyde et al. 2016; Maharachchikumbura et al. 2016; Luo et al. 2019; Dong et al. 2020; Calabon et al. 2022; Wijayawardene et al. 2022). Sordariomycetes is a prominent class within Ascomycota, encompassing a wide variety of fungi (Luo et al. 2019; Calabon et al. 2022; Yang et al. 2023). In freshwater environments, Sordariomycetes stands out as a significant fungal group, playing a pivotal role in ecosystems. This class is renowned for its production of bioactive compounds (e.g., penicillins, tetracyclines, macrolides, aminoglycosides, and cephalosporins) (Poch et al. 1992; Jones et al. 2014; Wright et al. 2014; Calabon et al. 2023).

*Aquapteridospora* was initially introduced and classified within the Diaporthomycetidae genera *incertae sedis*, based on morphological and phylogenetic analyses by Yang et al. (2015). *Aquapteridospora*, with *A.lignicola* as the type species, is characterized by polyblastic, sympodial, denticles conidiogenous cells and fusiform, with pale to dark brown central cells and subhyaline end cells, with or without sheath conidia. Furthermore, Hyde et al. (2021a) introduced the family Aquapteridosporaceae to accommodate *Aquapteridospora* and placed this family in order Distoseptisporales based on divergence estimates, morphological characters, and phylogenetic analyses.

*Aquapteridospora* is a hyphomycetous genus that are commonly found in freshwater habitats, but only a few terrestrial species, such as *A.bambusinum* (≡*Pleurophragmiumbambusinum*) was collected from dead culms of bamboo (Yang et al. 2015; Dai et al. 2017; Luo et al. 2019; Bao et al. 2021; Dong et al. 2021; Ma et al. 2022; Peng et al. 2022). These fungi play an important role in the decomposition of organics and nutrient cycling in aquatic environments (Hyde et al. 2016; Luo et al. 2018). In recent years, an increasing number of species in *Aquapteridospora* have been described and documented, including *A.aquatica*, *A.bambusinum*, *A.fusiformis*, *A.hyalina*, *A.jiangxiensis* and *A.lignicola* (Yang et al. 2015; Luo et al. 2019; Bao et al. 2021; Dong et al. 2021; Ma et al. 2022; Peng et al. 2022).

During an investigation of freshwater fungal diversity on the Tibetan Plateau, six collections possessing morphological characteristics that fit within the genus *Aquapteridospora* were collected. In particular, their morphological characteristics revealed that these collections were morphologically different from the other species in *Aquapteridospora*. In addition, phylogenetic analyses of a combined LSU, *TEF*1-α and ITS sequence data show that our new collections belong to distinct clades, which are distinct from other species in *Aquapteridospora*. Therefore, three new species *viz. Aquapteridosporalinzhiensis*, *A.submersa* and *A.yadongensis* are introduced, as well as details of asexual morphs being described, and justifications for establishing these new species are provided in this study.

## ﻿Materials and methods

### ﻿Collection, morphological examination and isolation

Submerged decaying wood samples were collected from freshwater habitats in southeast Xizang, China. Fresh specimens were studied following the methods of Senanayake et al. (2020). Microscopic structures were examined by using a stereomicroscope (SteREO Discovery.V12, Carl Zeiss Microscopy GmBH, Germany), photographed by using a Nikon ECLIPSE 80i compound microscope fitted with a Nikon DS-Ri2 digital camera, and measured by using the Tarosoft (R) Image Frame Work program. Illustrated figures were processed by using Adobe Photoshop CS6 version 10.0 software (Adobe Systems, San Jose, CA, USA).

Single spore isolation was performed on potato dextrose agar (PDA) plates following the methods described in Senanayake et al. (2020). Fungal herbarium specimens and axenic living cultures were deposited in the Herbarium of Cryptogams of the Kunming Institute of Botany, Chinese Academy of Sciences (KUN-HKAS) and Kunming Institute of Botany Culture Collection (KUNCC), Kunming, China. Faceoffungi and Index Fungorum numbers of novel species were registered (Jayasiri et al. 2015, http://www.indexfungorum.org/Names/Names.asp).

### ﻿DNA extraction, PCR amplification, and sequencing

Fresh mycelia were scraped off from colonies on PDA plates and transferred to a 1.5-ml microcentrifuge tube using a sterilized lancet for genomic DNA extraction. The TOLOBIO Plant Genomic DNA Extraction Kit, Shanghai Co. Ltd. P.R. China was used to extract fungal genomic DNA, following the protocols in the manufacturer’s instructions. The DNA polymerase chain reaction (PCR) amplifications were performed by using primer pairs as follows: ITS5/ITS4 for internal transcribed spacer rDNA region and covered 5.8S ribosomal (ITS); LR0R/LR5 for the nuclear ribosomal large subunit 28S rDNA gene (LSU), and *TEF*1-983F/*TEF*1-2218R for *TEF*1-*α* (Vilgalys and Hester 1990; White et al. 1990). DNA template was carried out in 25 μL reaction volume containing 21 μL of 1 × Power Taq PCR Master Mix, 1 μL of each primer (10 μL stock) and 2 μL of genomic DNA template. Amplifications were carried out by using the BioTeke GT9612 thermocycler (Beijing City, China). The PCR amplification conditions for ITS and LSU consisted of initial denaturation at 98 °C for 3 minutes, followed by 35 cycles of denaturation at 98 °C for 20 seconds, annealing at 53 °C for 10 seconds, extension at 72 °C for 20 seconds, final extension at 72 °C for 5 minutes; the PCR amplification conditions for *TEF*1-*α* consisted of initial denaturation at 98 °C for 3 minutes, followed by 35 cycles of denaturation at 98 °C for 20 seconds, annealing at 64 °C for 10 seconds, extension at 72 °C for 20 seconds, final extension at 72 °C for 5 minutes. PCR products were visualized by using 1% agarose gel electrophoresis stained with ethidium bromide and distinct bands were checked in Gel documentation system (Compact Desktop UV Transilluminator analyzer GL-3120). The PCR products were sequenced by Tsingke Company, Beijing, P.R. China.

### ﻿Phylogenetic analyses

The sequences were uploaded in GenBank database (http://www.ncbi.nlm.nih.gov/blast/) to search for similar taxa. Sequences generated from the LSU, *TEF*1-α and ITS gene regions were carefully verified before further analyses. The new sequences were submitted to GenBank, and the strain information used in this paper was provided in Table 1. Multiple sequence alignments were aligned with MAFFT v.7 (Katoh and Standley 2016) http://mafft.cbrc.jp/alignment/server/index.html] and dataset was trimmed by TrimAlv.1.3 using the gappyout option (http://phylemon.bioinfo.cipf.es/utilities.html) (Capella-Gutierrez et al. 2009). A combined sequence dataset was performed with the SquenceMatrix v.1.7.8 (Vaidya et al. 2011).

**Table 1.** Strains used for phylogenetic analyses and their corresponding GenBank numbers. The newly generated sequences are in cells with light grey shading and the type strain are in bold font.

Species	Voucher number	GenBank accession number			Reference
		LSU	ITS	*TEF*1-α	
***Aquapteridosporaaquatica***	**MFLUCC 17-2371**	**MW287767**	**MW286493**	/	Dong et al. (2021)
***A.bambusinum***	**MFLUCC 12-0850**	**KU863149**	**KU940161**	**KU940213**	Dai et al. (2017)
*A.bambusinum*	MFLUCC_21_0027	MZ412526	MZ412514	MZ442688	Bao et al. (2021)
***A.hyalina***	**GZCC 22-0072**	**ON527945**	**ON527937**	**ON533681**	Ma et al. (2022)
*A.hyalina*	GZCC 22-0073	ON527948	ON527940	ON533684	Ma et al. (2022)
***A.jiangxiensis***	**JAUCC 3008**	**MZ871502**	**MZ871501**	**MZ855767**	Peng et al. (2022)
***A.fusiformis***	**MFLUCC 18-1606**	**MK849798**	**MK828652**	**MN194056**	Luo et al. (2019)
***A.lignicola***	**MFLUCC 15-0377**	**KU221018**	**MZ868774**	**MZ892980**	Yang et al. (2015)
***A.linzhiensis***	**KUNCC 10420**	**OQ970576**	**OP626343**	**OR597592**	This study
*A.linzhiensis*	KUNCC 10444	OQ970575	OQ847781	OR597591	This study
***A.submersa***	**KUNCC 10446**	**OQ970579**	**OQ847783**	**OR597595**	This study
*A.submersa*	KUNCC 10449	OQ970580	OQ970557	OR597596	This study
***A.yadongensis***	**KUNCC 10445**	**OQ970577**	**OQ847782**	**OR597593**	This study
*A.yadongensis*	KUNCC 10448	OQ970578	OQ970556	OR597594	This study
***Distoseptisporaatroviridis***	**GZCC 20-0511**	**MZ868763**	**MZ868772**	**MZ892978**	Yang et al. (2021)
***D.bambusae***	**MFLUCC 20-0091**	**MT232718**	**MT232713**	**MT232880**	Sun et al. (2020)
*D.euseptata*	MFLU 20-0568	MW081545	MW081540	MW084994	Li et al. (2021)
***D.fusiformis***	**GZCC 20-0512**	**MZ868764**	**MZ868773**	**MZ892979**	Yang et al. (2021)
***D.guizhouensis***	**GZCC 21-0666**	**MZ474869**	**MZ474868**	**MZ501610**	Hyde et al. (2021b)
***D.hyalina***	**MFLUCC 17-2128**	**MZ868760**	**MZ868769**	**MZ892976**	Yang et al. (2021)
*D.multiseptata*	MFLU 17-0856	MF077555	MF077544	MF135652	Yang et al. (2018)
***D.rayongensis***	**MFLUCC 18-0415**	**MH457137**	**MH457172**	**MH463253**	Hyde et al. (2020)
*D.rayongensis*	MFLUCC 18-0417	MH457138	MH457173	MH463254	Hyde et al. (2020)
***D.rostrata***	**MFLUCC 16-0969**	**MG979766**	**MG979758**	**MG988424**	Luo et al. (2018)
***D.saprophytica***	**MFLUCC 18-1238**	**MW287780**	**MW286506**	**MW396651**	Dong et al. (2021)
***D.verrucosa***	**GZCC 20-0434**	**MZ868762**	**MZ868771**	**MZ892977**	Yang et al. (2021)
***D.xishuangbannaensis***	**KUMCC 17-0290**	**MH260293**	**MH275061**	**MH412768**	Tibpromma et al. (2018)
***D.yunnansis***	**MFLUCC 20-0153**	**MW081546**	**MW081541**	**MW084995**	Li et al. (2021)
***Pseudostanjehughesiaaquitropica***	**MFLUCC 16-0569**	**MF077559**	**MF077548**	**MF135655**	Yang et al. (2018)
***P.lignicola***	**MFLUCC 15-0352**	**MK849787**	**MK828643**	**MN194047**	Luo et al. (2019)
***Sporidesmiumdulongense***	**MFLUCC 17-0116**	**MH795817**	**MH795812**	**MH801191**	Luo et al. (2019)
***S.lageniforme***	**DLUCC 0880**	**MK849782**	**MK828640**	**MN194044**	Luo et al. (2019)
***S.pyriformatum***	**MFLUCC 15-0620**	**KX710141**	**KX710146**	**MF135662**	Hyde et al. (2016)
*S.thailandense*	MFLUCC 15-0617	MF077561	MF077550	MF135657	Yang et al. (2018)
***S.thailandense***	**MFLUCC 15-0964**	**MF374370**	**MF374361**	**MF370957**	Zhang et al. (2017)
***Myrmecridiumaquaticum***	**MFLUCC 15-0366**	**MK849804**	/	/	Luo et al. (2019)
*M.aquaticum*	S-1158	MK849803	MK828656	MN194061	Luo et al. (2019)
***M.banksiae***	**CBS 132536**	**JX069855**	**JX069871**	/	Crous et al. (2012)
*M.schulzeri*	CBS 100.54	EU041826	EU041769	/	Arzanlou et al. (2007)

**Figure 1.**
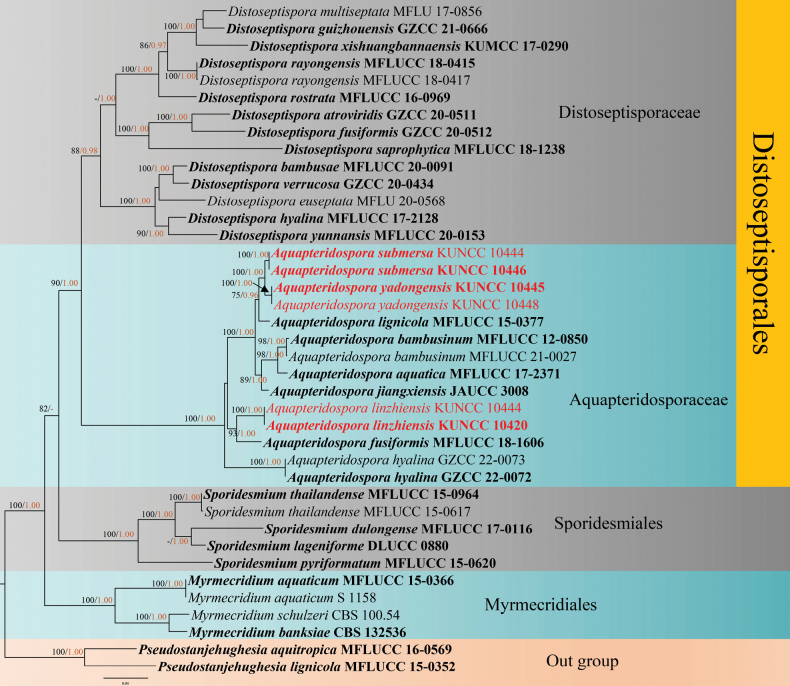
Maximum likelihood (ML) tree is based on combined LSU, *TEF*1-α and ITS sequence data. ML bootstrap support values equal to or greater than 70% and Bayesian posterior probabilities (PP) equal to or greater than 0.95 given above the nodes, shown as “ML/PP”. The tree is rooted with *Pseudostanjehughesiaaquitropica* (MFLUCC 16-0569) and *P.lignicola* (MFLUCC 15-0352). New species are indicated in red and type strains are in bold.

Maximum likelihood (ML) analysis was performed by RAxML-HPC2 v.8.2.12 (Stamatakis 2014) in the CIPRES Science Gateway web server (http://www.phylo.org/portal2) by using 1,000 rapid bootstrap replicates and the GTRGAMMA+I model. Bootstrap support values for ML equal to or greater than 75% were given above the nodes in the phylogenetic tree (Fig. 1). The model of evolution for the Bayesian inference (BI) analysis was performed by using MrModeltest v2.3 (Nylander 2004). GTR+I+G was selected as the best-fitting model for LSU, *TEF*1-α and ITS dataset. The Markov chain Monte Carlo sampling (BMCMC) was carried out to assess posterior probabilities (PP) by using MrBayes v.3.2.7 (Ronquist et al. 2012). Six simultaneous Markov chains were run for random trees for 1,000,000 generations, and trees were sampled every 200^th^ generation. Bayesian posterior probabilities (PP) equal to or greater than 0.95 were given above the nodes in the phylogenetic tree (Fig. 1). Phylograms were visualized by using FigTree v1.4.0 (Rambaut 2012) and rearranged in Adobe Photoshop CS6 software (Adobe Systems, USA). The new sequences were deposited in GenBank (Table 1), and the final alignments and phylogenetic tree were registered in TreeBASE under the submission ID: 30133 (http://www.treebase.org/).

## ﻿Results

### ﻿Phylogenetic analyses

The concatenated sequence dataset of LSU, *TEF*1-α and ITS, comprised 39 strains with *Pseudostanjehughesiaaquitropica* (MFLUCC 16-0569) and *P.lignicola* (MFLUCC 15-0352) as the outgroup taxa (Fig. 1). The datasets contained 2,168 characters including gaps after alignments (LSU: 1–763 bp, -*α* = 764–1,660 bp, ITS: 1,661–2,168 bp). The RAxML analysis of the combined datasets yielded a best scoring tree with a final ML optimization likelihood value of -15404.143090. The aligned sequences matrix comprised 849 distinct alignment patterns with 6.45% of undetermined characters or gaps. Estimated base frequencies were as follows: A = 0.229844, C = 0.282249, G = 0.282387, T = 0.205520, with substitution rates AC = 0.921073, AG = 2.039438, AT = 1.172967, CG = 0.817703, CT = 5.518393, GT = 1.000000; gamma distribution shape parameter α = 0.0010000000. The tree topologies of combined sequence data obtained from ML and BI analyses were not significantly different (Fig. 1).

The phylogenetic analyses showed that our six strains nested within the genus *Aquapteridospora* represent three species. Two strains of *A.linzhiensis* (KUNCC 10420 and KUNCC 10444) formed a well resolved subclade sister to *A.fusiformis* (93% ML/1.00 PP support); while strains of *A.yadongensis* (KUNCC 10445 and KUNCC 10448) formed a distinct subclade sister to *A.submersa* (KUNCC 10446 and KUNCC 10449) with a high support (100% ML/1.00 PP) and clustered with *A.lignicola* (MFLUCC 15-10377) with a significant support (75% ML/0.96 PP) (Fig. 1).

### ﻿Taxonomy

#### ﻿ Aquapteridospora linzhiensis

Taxon classificationFungiDistoseptisporalesAquapteridosporaceae

R.J. Xu, Q. Zhao & Boonmee sp. nov.

DE0C6450-5534-5F55-8F32-A7CBE4CDB733

Index Fungorum: IF901109

Facesoffungi Number: FoF14348

Fig. 2

##### Etymology.

Referring to the location “Linzhi City, China” where the holotype of this fungus was collected.

##### Holotype.

HKAS 128991.

##### Description.

***Saprobic*** on decaying wood submerged in freshwater. **Sexual morph**: Undetermined. **Asexual morph: *Colonies*** on the natural substrate effuse, hairy, pale brown to brown, scattered or in small groups. ***Mycelium*** mostly superficial, consisting of branched, septate, smooth, pale brown to brown hyphae. ***Conidiophores*** 113–210 × 4–6 μm (x̄ = 162 × 4 μm, n = 15), macronematous, mononematous, solitary or 2–3 group, erect, straight or slightly flexuous, simple, unbranched, smooth, cylindrical, 6–12-septate, brown at the base, pale brown towards apex. ***Conidiogenous cells*** polyblastic, monoblastic, terminal, becoming intercalary, cylindrical, pale brown, integrated, with several sympodial proliferations, conspicuous denticles, bearing tiny, protuberant, circular scars. ***Conidia*** 10–14 × 5–6 μm (x̄ = 12 × 6 μm, n = 25), solitary or acropleurogenous, fusiform or elliptical, smooth, 2-septate, truncate at base, dark brown in central cells and subhyaline at end cells, guttulate. Conidial secession schizolytic.

**Figure 2.**
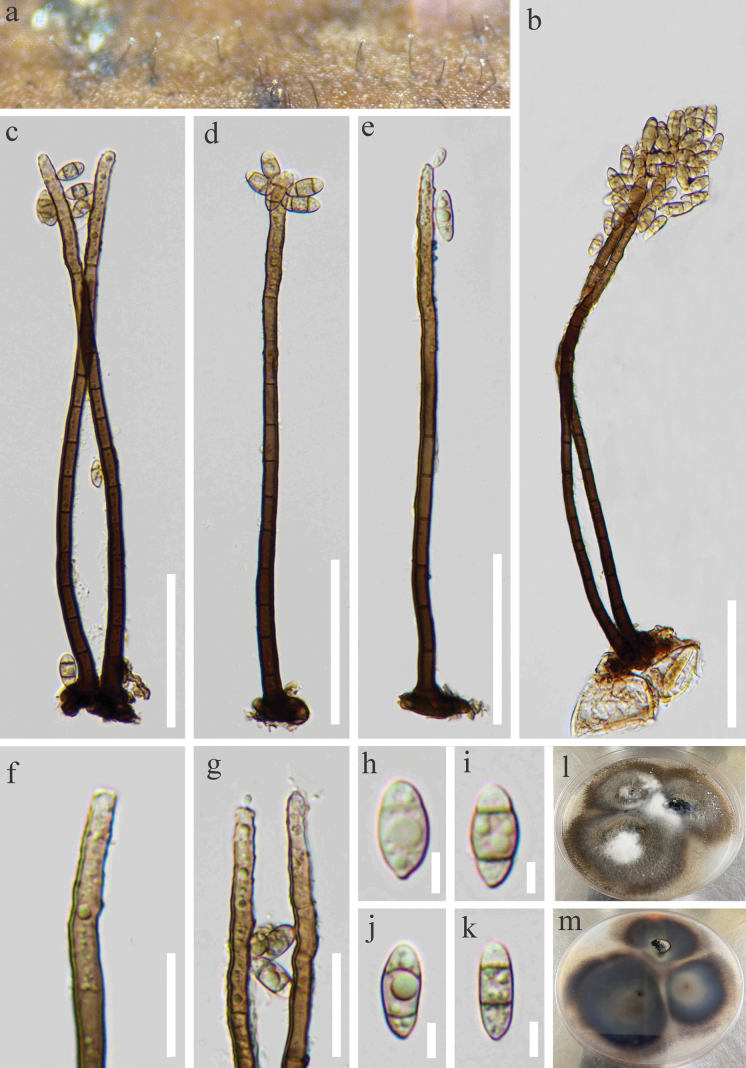
*Aquapteridosporalinzhiensis* (HKAS 128991, holotype) **a** colonies on the substratum **b–e** conidiophores, conidiogenous cells with conidia **f, g** conidiogenous cells with developmental conidia **h–k** conidia **l, m** culture on PDA. Scale bars: 50 μm (**b–e**); 20 μm (**f, g**); 10 μm (**h–k**).

##### Culture characteristics.

Conidia were germinated on PDA within 48 hours. Germ tubes produced from each end. Colonies grown on PDA, circular, flat, superficial, dark brown from above, reverse-side brown in the centre, with greyish white near the edge.

##### Material examined.

China, Xizang, Linzhi City, Motuo County, on submerged decaying wood, 1675 msl, 29°10'56"N, 95°8'53"E, 11 July 2022, R.J. Xu, XK-33–3 (HKAS 128991, holotype), ex-type living culture (KUNCC 10420). Xizang, Linzhi City, Motuo County, Gelin Village, on submerged decaying wood, 1143 msl, 29°1'43"N, 94°48'5.7"E, 12 July 2022, R.J. Xu, XK-32, (HKAS 128990), living culture (KUNCC 10444).

##### Notes.

Phylogenetic analyses show that *Aquapteridosporalinzhiensis* (KUNCC 10420 and KUNCC 10444) clustered into a distinct subclade and sister to *A.fusiformis* (MFLUCC 18-1606) with bootstrap support (93% ML/1.00 PP, Fig. 1). However, *A.linzhiensis* differs from *A.fusiformis* in having obvious, guttulate conidia and less septate on maturity (2-septate *vs.* 3–4-septate) (Luo et al. 2019). Additionally, comparisons of ITS sequences demonstrate a 6.7% (39/586 bp, excluding gaps) difference between *A.linzhiensis* and *A.fusiformis*Jeewon and Hyde (2016). Therefore, *A.linzhiensis* was identified as a new species supported with both morphological and phylogenetic evidences.

#### ﻿ Aquapteridospora yadongensis

Taxon classificationFungiDistoseptisporalesAquapteridosporaceae

R.J. Xu, Q. Zhao & Boonmee sp. nov.

A0FCBE8F-17CB-5340-B167-5E5F633EC92A

Index Fungorum: IF901110

Facesoffungi Number: FoF14349

Fig. 3

##### Etymology.

Referring to the location “Yadong County, China” where the holotype of this fungus was collected.

##### Holotype.

HKAS 128992.

##### Description.

***Saprobic*** on decaying wood submerged in freshwater. **Sexual morph**: Undetermined. **Asexual morph: *Colonies*** on the natural substrate effuse, hairy, pale brown to brown, scattered or in small groups, usually retiform. ***Mycelium*** mostly superficial, consisting of branched, septate, smooth, pale brown to brown hyphae. ***Conidiophores*** 440–856 × 4–6 μm (x̄ = 581 × 5 μm, n = 20), macronematous, mononematous, solitary, erect, straight or slightly flexuous, unbranched, smooth, cylindrical, multi-septate, tapering towards apex, brown to pale brown, slightly constricted at some septa. ***Conidiogenous cells*** polyblastic, monoblastic, terminal, becoming intercalary, cylindrical, pale brown, integrated, denticles, bearing tiny, protuberant, circular scars. ***Conidia*** 14–20 × 4–7 μm (x̄ = 17 × 5 μm, n = 30), acropleurogenous, fusiform, smooth, 3-septate, rounded at apex, truncate at base, dark brown in central cells and light at end cells. Conidial secession schizolytic.

**Figure 3.**
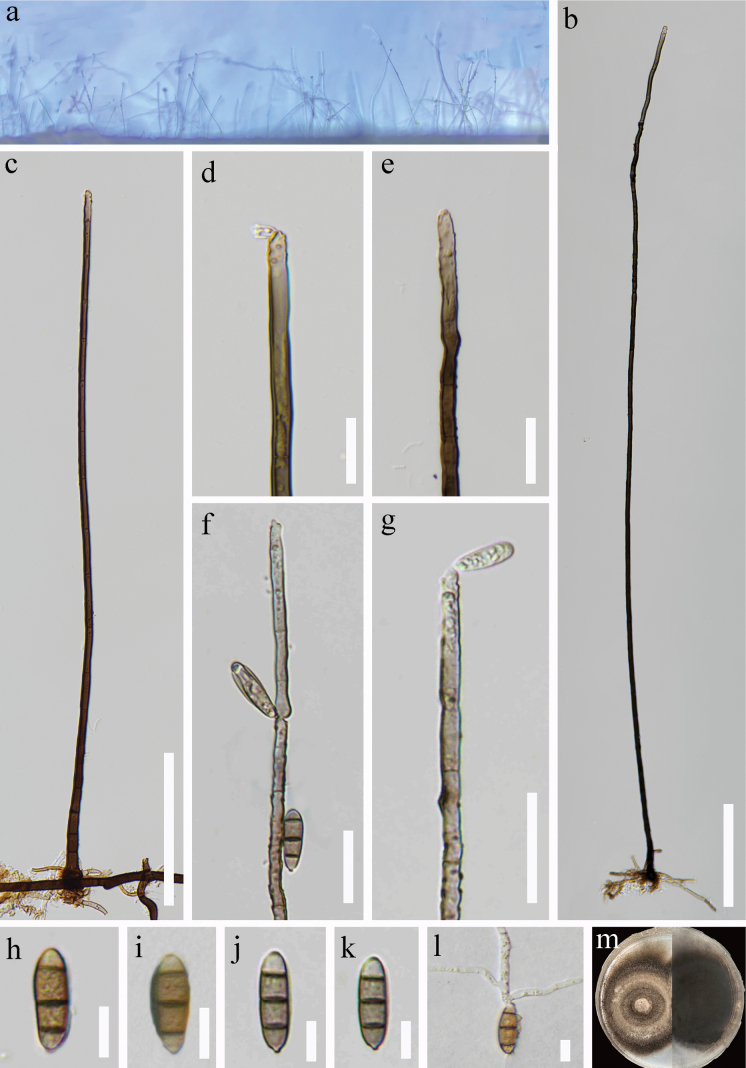
*Aquapteridosporayadongensis* (HKAS 128992, holotype) **a** colonies on the substratum **b, c** conidiophore and conidiogenous cell **d-g** conidiogenous cells with developmental conidia **h–k** conidia **l** germinating conidium **m** culture on PDA. Scale bars: 100 μm (**b, c**); 20 μm (**d, g**); 10 μm (**h–l**).

##### Culture characteristics.

Conidia were germinated on PDA within 48 hours. Germ tubes produced from each end. Colonies grown on PDA, regular concentric circles, flat, superficial, with dense mycelium at around, grey brown from above, dark brown from below.

##### Material examined.

China, Xizang, Shigatse City, Yadong County, on submerged decaying wood, 3061 msl, 27°21'11"N, 88°58'10"E, 01 July 2022, R.J. Xu, LTS-20 (HKAS 128992, holotype), ex-type living culture (KUNCC 10445). Xizang, Shigatse City, Dingjie County, on submerged decaying wood, 3042 msl, 27°53'8.7"N, 87°27'36"E, 05 July 2022, L.T. Shun, LTS-20–1, (HKAS 128993), living culture (KUNCC 10448).

##### Notes.

*Aquapteridosporayadongensis* possess its conidial characteristics that fit with *Aquapteridospora* (Yang et al. 2015). In phylogenetic analyses, *A.yadongensis* formed a distinct lineage close to *A.submersa* with high bootstrap support (100% ML/1.00 PP, Fig. 1). A comparison of ITS nucleotide shows that *A.yadongensis* (KUNCC 10445) differs from *A.submersa* (KUNCC 10446) in 10/572 bp (1.8%, excluding gap), a comparison of *TEF*1-α nucleotide shows that *A.yadongensis* (KUNCC 10445) differs from *A.submersa* (KUNCC 10446) in 8/821 bp (0.8%, excluding gap) (Jeewon and Hyde 2016). In addition, *A.yadongensis* differs from *A.submersa* in having narrower conidiophores (4–6 *vs.* 5–12 μm), while conidia of *A.submersa* have slightly constricted septa; the culture of *A.yadongensis* have regular concentric circles differing from *A.submersa* having pale mycelium in the centre. Furthermore, *A.yadongensis* differs from *A.lignicola* in having long conidiophores (440–856 *vs.* 70–200 μm) and conidia without a conspicuous sheath (Yang et al. 2015).

#### ﻿ Aquapteridospora submersa

Taxon classificationFungiDistoseptisporalesAquapteridosporaceae

R.J. Xu, Q. Zhao & Boonmee sp. nov.

9D3EEDA1-9E38-58BC-811D-EC1464144577

Index Fungorum: IF901111

Facesoffungi Number: FoF14350

Fig. 4

##### Etymology.

Referring to the fungus’s habitat “decaying wood submerged in freshwater habitats”.

##### Holotype.

HKAS 128980.

##### Description.

***Saprobic*** on decaying wood submerged in freshwater. **Sexual morph**: Undetermined. **Asexual morph: *Colonies*** on the natural substrate effuse, glistening, pale brown to brown, scattered or in small groups. ***Mycelium*** mostly superficial, consisting of branched, septate, smooth, pale brown to brown hyphae. ***Conidiophores*** 376–708 × 5–12 μm (x̄ = 451 × 7 μm, n = 20), macronematous, mononematous, solitary, erect, straight or slightly flexuous, unbranched, smooth, cylindrical, multi-septate, tapering towards apex, brown to pale brown. ***Conidiogenous cells*** polyblastic, monoblastic, terminal, becoming intercalary, cylindrical, pale brown, integrated, with several sympodial proliferations, conspicuous denticles, bearing tiny, protuberant, circular scars. ***Conidia*** 19–22 × 6–8 μm (x̄ = 21 × 7 μm, n = 20), solitary or acropleurogenous, fusiform, smooth, 2–3-septate, rounded at apex, truncate at base, slightly constricted at septa, hyaline when young, sub-hyaline to pale brown when mature, two big guttulate when young. Conidial secession schizolytic.

**Figure 4.**
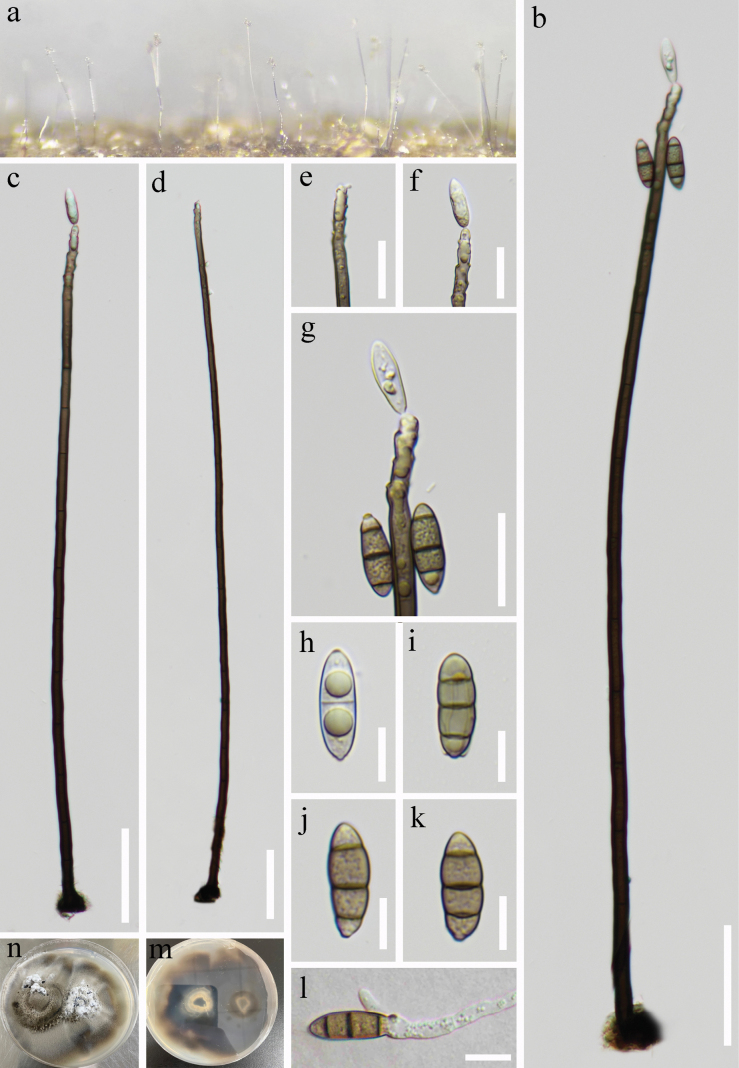
*Aquapteridosporasubmersa* (HKAS 128980, holotype) **a** colonies on the substratum **b–d** conidiophores, conidiogenous cells with conidia **e–g** conidiogenous cells with developmental conidia **h–k** conidia **l** germinating conidium **m, n** culture on PDA. Scale bars: 50 μm (**b–d**); 20 μm (**e–g**); 10 μm (**h–l**).

##### Culture characteristics.

Conidia were germinated on PDA within 48 hours. Germ tubes produced from each end. Colonies grown on PDA, circular, flat, superficial, raised, with dense, pale mycelium in the centre. Grey brown from above, dark brown from below.

##### Material examined.

China, Xizang, Linzhi City, Motuo County, on submerged decaying wood, 677 msl, 29°19'43"N, 95°21'19"E, 13 July 2022, R.J. Xu, LJN-15 (HKAS 128980, holotype), ex-type living culture (KUNCC 10446). Xizang, Linzhi City, Motuo County, Gelin Village, on submerged decaying wood, 677 msl, 29°19'43"N, 95°21'19"E, 12 July 2022, R.J. Xu, LJN-15–5, (HKAS 128981), living culture (KUNCC 10444).

##### Notes.

Phylogenetic analyses show that *Aquapteridosporasubmersa* (KUNCC 10446, KUNCC 10444), formed a sister grouped with *A.yadongensis* (KUNCC 10445 and KUNCC 10488) and was close to *A.lignicola* (MFLUCC 15-0377) with 75% ML/0.96 PP, Fig. 1. However, the comparison of conidial characteristics and nucleotides shows that *A.submersa* differs from *A.yadongensis* (see the notes of *A.yadongensis*). Indeed, *A.submersa* differs from *A.lignicola* in having long conidiophores (376–708 *vs.* 70–200 μm) and conidia without a conspicuous sheath (Yang et al. 2015). *Aquapteridosporasubmersa* is introduced here as a new species.

## ﻿Discussion

Species of *Aquapteridospora* are morphologically unique in the taxonomic characteristics, especially in the features of the conidiophores and conidia (Table 2). In most species, the conidia are fusiform and pigmented, featuring brown to dark brown central cells and subhyaline end cells. However, some species exhibit conidia with a distinct sheath, such as *A.aquatica*, *A.jiangxiensis* and *A.lignicola* (Yang et al. 2015; Dong et al. 2021; Peng et al. 2022). Additionally, a few species are characterized by hyaline to sub-hyaline conidia, as observed in *A.hyalina* (Ma et al. 2022). In addition, the length of conidiophores in species of *Aquapteridospora* varies significantly. Most species have conidiophores ranging in length from 70 to 305 μm, as observed in species like *A.aquatica*, *A.fusiformis*, *A.hyalina*, *A.jiangxiensis*, *A.lignicola* and *A.linzhiensis* (Yang et al. 2015; Luo et al. 2019; Dong et al. 2021; Ma et al. 2022; Peng et al. 2022), a few species exhibit conidiophores exceeding 400 μm in length, with the longest reaching 856 μm. This is the case for species such as *A.bambusinum*, *A.yadongensis* and *A.submersa* (Bao et al. 2021, this study).

**Table 2.** Synopsis of known species in *Aquapteridospora*.

Species	Conidiophores (μm)	Conidiogenous cells (μm)	Conidia (μm)	Host	Habitat	Distribution	Reference
*Aquapteridosporaaquatica*	125–215 × 3–5	10–85 × 4–5.5, Polyblastic, terminal, intercalary, denticles	19–27.5 × 5–7.5, acropleurogenous, solitary, olivaceous or brown in the middle cells, fusiform, 3-septate, gelatinous, thin sheath	Unidentified, submerged wood	Freshwater	Thailand	Dong et al. (2021)
*A.bambusinum*	615–715 × 9–13	Polyblastic, sympodial, denticulate, integrated, terminal	15–18 × 5.5–7, acrogenous, solitary, pale brown to dark brown, ellipsoid to fusiform, 3-septate, straight	Unidentified, submerged wood	Freshwater	Thailand	Bao et al. (2021)
*A.fusiformis*	(88–) 134–188 × 5–7	Polyblastic, terminal, intercalary, sympodial proliferations	14–18 × 5–7, solitary, brown to dark brown in central cells and subhyaline at end cells, fusiform, 3–4-septate,	Unidentified, submerged wood	Freshwater	China	Luo et al. (2019)
*A.hyalina*	68–130 × 4.5–6.5	25–62 × 4–6.5, polyblastic, monoblastic, denticles	17–28 × 4–6, acropleurogenous, solitary, sub-hyalina to pale brown, fusiform, 1–3-septate,	Unidentified, submerged wood	Freshwater	China	Ma et al. (2022)
*A.jiangxiensis*	78–305 × 4–7	20–68 × 4–6, integrated, terminal, intercalary	20–25 × 6–7.5, acrogenous or lateral, dark brown to black, fusiform to subclavate, 3-septate, sometimes with a sheath	Unidentified, submerged wood	Freshwater	China	Peng et al. (2022)
*A.lignicola*	70–200 × 4–7	14.5–30 × 4.5–7.5, polyblastic, terminal, intercalary	15–24 × 6–8, solitary, acropleurogenous, with pale to dark brown central cells and subhyaline end cells, fusiform, 3-septate, with a conspicuous sheath	Unidentified, submerged wood	Freshwater	Thailand	Yang et al. (2015)
*A.linzhiensis*	113–210 × 4–6	Polyblastic, terminal, intercalary, denticles	10–14 × 5–6, solitary or acropleurogenous, dark brown in central cells and subhyaline at end cells, fusiform or elliptical, 2-septate, guttulate	Unidentified, submerged wood	Freshwater	China	This study
*A.yadongensis*	440–856 × 4–6	Polyblastic, monoblastic, terminal, intercalary, denticles	14–20 × 4–7, acropleurogenous, dark brown in central cells and subhyaline at end cells, fusiform, 3-septate	Unidentified, submerged wood	Freshwater	China	This study
*A.submersa*	376–708 × 5–12	Polyblastic, monoblastic, terminal, intercalary, denticles	19–22 × 6–8, solitary or acropleurogenous, hyaline when young, sub-hyaline to pale brown when mature, fusiform, 2–3-septate, two big guttulate when young	Unidentified, submerged wood	Freshwater	China	This study

Molecular phylogenetic analyses play a crucial role in elucidating the classification of hyphomycetous fungi (Dhanasekaran et al. 2006; Tekpinar and Kalmer 2019). *Pleurophragmiumbambusinum* was initially described by Dai et al. (2017), and was previously assigned to Sordariomycetes*incertae sedis* based on its morphological characteristics. According to the phylogenetic analysis conducted by Dong et al. (2021), *P.bambusinum* was found to cluster within the *Aquapteridospora* clade with (100% ML/1.00 PP) support. However, their studies did not synonymize *P.bambusinum* under *Aquapteridospora* due to the ellipsoidal and conidia without a sheath, which indicate that it does not fit within the characteristics of *Aquapteridospora* species. Subsequently, Bao et al. (2021) transferred *P.bambusinum* to *Aquapteridospora* and synonymized *A.bambusinum* instead of *P.bambusinum*, based on both phylogeny and morphology.

The Tibetan Plateau is renowned for its distinctive biological diversity and extensive array of aquatic habitats, encompassing lakes, rivers, and wetlands, which provide sustenance for various fungal communities (Yao et al. 2019). While freshwater fungi play a crucial role in the ecosystem, they have remained understudied in this region, primarily due to the limited number of researchers focusing on freshwater fungi in the Tibetan Plateau. During our investigation into freshwater fungal diversity on the Tibetan Plateau, we introduced three new species within the genus *Aquapteridospora*, supported by both phylogenetic analysis and morphology. The discovery of these new species revealed the abundant fungal diversity in Tibetan Plateau and more scientific studies in this region are expected in the future.

## Supplementary Material

XML Treatment for Aquapteridospora linzhiensis

XML Treatment for Aquapteridospora yadongensis

XML Treatment for Aquapteridospora submersa

## ﻿Additional information

### Author ORCIDs

Rong-Ju Xu https://orcid.org/0000-0002-3968-8442

Jun-Fu Li https://orcid.org/0009-0008-6088-2072

De-Qun Zhou https://orcid.org/0009-0009-3459-3186

Saranyaphat Boonmee https://orcid.org/0000-0001-5202-2955

Qi Zhao https://orcid.org/0000-0001-8169-0573

Ya-Ya Chen https://orcid.org/0000-0002-8293-168X

### Data availability

All of the data that support the findings of this study are available in the main text.
